# Influence of Exercise and Genistein to Mitigate the Deleterious Effects of High-Fat High-Sugar Diet on Alzheimer’s Disease-Related Markers in Male Mice

**DOI:** 10.3390/ijms25169019

**Published:** 2024-08-20

**Authors:** Juhi Shah, Tyler Orosz, Avneet Singh, Savan Parameshwar Laxma, Rachel E. Gross, Nicholas Smith, Spencer Vroegop, Sydney Sudler, James T. Porter, Maria Colon, Lauren Jun, Jeganathan R. Babu, Minsub Shim, Thomas L. Broderick, Layla Al-Nakkash

**Affiliations:** 1 Arizona College of Osteopathic Medicine, Midwestern University, 19555 N. 59th Avenue, Glendale, AZ 85308, USAtyler.orosz@midwestern.edu (T.O.); savan.parameshwarlax@midwestern.edu (S.P.L.); rachel.gross@midwestern.edu (R.E.G.); spencer.vroegop@midwestern.edu (S.V.); sydney.sudler@midwestern.edu (S.S.); 2 Department of Basic Sciences, Ponce Research Institute, Ponce Health Sciences University, Ponce 00732, Puerto Rico; jporter@psm.edu (J.T.P.); macolon@psm.edu (M.C.); 3 Department of Nutritional Sciences, Auburn University, Auburn, AL 36849, USA; 4 Department of Biochemistry, College of Graduate Studies, Midwestern University, 19555 N. 59th Avenue, Glendale, AZ 85308, USA; 5 Department of Physiology, College of Graduate Studies, Midwestern University, 19555 N. 59th Avenue, Glendale, AZ 85308, USA

**Keywords:** high-fat diet, sucrose, genistein, exercise, brain, Alzheimer’s disease

## Abstract

The prevalence of obesity and related consequences, including insulin resistance and Alzheimer’s-like neuropathology, has increased dramatically. Contributing to this prevalence is the shift in lifestyle preference away from wholesome foods and exercise to the Western-style diet and sedentarism. Despite advances in drug development, a healthy diet and regular exercise remain the most effective approaches to mitigating the unwanted sequelae of diet-induced obesity on brain health. In this study, we used the high-fat high-sugar (HFHS) mouse model of neurodegeneration to examine the effects of exercise training (HFHS+Ex), genistein treatment (HFHS+Gen), and combination treatment (HFHS+Ex+Gen) on proteins relating to neurodegeneration in the brain of male mice. After a period of 12 weeks, as expected, HFHS feeding increased body weight, adipose tissue weight, and systemic plasma inflammation (TNF-α) compared to lean mice fed a standard diet. HFHS feeding also increased protein expression of brain markers of insulin resistance (pGSK-3β, p-IR), apoptosis (caspase 3), early neurofibrillary tangles (CP13), and amyloid-beta precursor (CT20). Compared to HFHS mice, Ex decreased body weight, plasma TNF-α, and expression of pGSK-3β, caspase 3, CP13, amyloid-β precursor (22c11), and ADAM10. Treatment with Gen was equally protective on these markers and decreased the expression of p-IR. Combination treatment with Ex and Gen afforded the greatest overall benefits, and this group exhibited the greatest reduction in body and adipose tissue weight and all brain markers, except for 22c11 and ADAM10, which were decreased compared to mice fed an HFHS diet. In addition, levels of 4G8, which detects protein levels of amyloid-β, were decreased with combination treatment. Our results indicate that exercise training, genistein supplementation, or combination treatment provide varying degrees of neuroprotection from HFHS feeding-induced Alzheimer’s pathology. Future perspectives could include evaluating moderate exercise regimens in combination with dietary supplementation with genistein in humans to determine whether the same benefits translate clinically.

## 1. Introduction

In recent decades, there has been an unpreceded rise in the incidence and prevalence of insulin resistance, obesity, type 2 diabetes (T2DM), and cardiovascular diseases [1,2]. This is attributed, in part, to the introduction of highly available ultra-processed foods in the form of saturated fats, refined grains, and sugars. As a result, the chronic consumption of these energy-rich foods, also known as the “Western diet”, has increased dramatically at the expense of the intake of healthy wholesome foods [3]. In addition to the well-documented adverse effects on overall health, emerging evidence has shown that excessive intake of energy-rich foods is associated with detrimental consequences on brain function. Chronic consumption of high-fat high-sugar (HFHS) diet in humans and experimental models is linked to impaired cognitive function and contributes to the development of neurodegenerative diseases, including Alzheimer’s disease (AD) [4,5,6,7,8,9,10,11]. Evidence indicates that dysregulated central insulin signaling from HFHS feeding is an underlying cause of brain function, memory loss, and dementia in AD [12,13,14].

With the epidemic of diet-induced obesity, T2DM, and increasing evidence of the deleterious effects of Western-type diets on brain health, there is a clear need for treatment options to mitigate the consequences of these metabolic disorders. The naturally occurring compound genistein, a phytoestrogen found in soy [15], has been shown to have health benefits in T2DM, severe obesity, and neurodegenerative diseases [16,17,18,19]. We have previously shown that treatment with genistein has beneficial effects in the ob/ob mouse model of severe obesity, hyperglycemia, and brain markers of AD [16,20]. Genistein decreased weight gain, improved glucose tolerance, increased the expression of both brain-derived neurotrophic factor (BDNF) and nerve growth factor (NGF), and decreased amyloid beta (Aβ) deposition [20]. More recently, we demonstrated that the increased Aβ and phosphorylation of tau loads induced by chronic HFHS feeding in female mice were mitigated by genistein treatment [21]. Other studies have also reported beneficial effects of genistein on the diabetic state and neural function [22,23,24,25,26]. Taken together, genistein has been shown to not only improve metabolic control in diabetes and obesity, but also increase neurotrophic factors and alleviate AD-related markers. 

In addition to the impact of obesogenic diets on the development of metabolic- and brain-related disorders, evidence indicates that physical inactivity and sedentary behavior are major contributors to chronic diseases [27,28,29]. Physical inactivity accelerates biological aging and is highly prevalent, with recent estimates from the Center for Disease Control indicating that roughly 25% of US adults are considered physically inactive [30]. On the other hand, regular exercise is known to delay the onset of chronic diseases and decrease the risk of neurodegenerative diseases [4,31,32,33]. Hence, the value of exercise as an alternate non-pharmacological approach of care options for alleviating symptoms of diabetes and provision of overall health benefits is recognized. While there is substantial evidence indicating that engaging in various exercise strategies decreases the risk of neurodegenerative diseases and AD [5,34,35,36,37], a recent systematic review indicated that physical activity does not modulate the pathophysiology in certain patients with AD [38]. However, in experimental models, exercise has been shown to prevent Aβ deposition in the brain [39,40,41,42,43,44], and offers some protection on Aβ accumulation and apoptosis by increasing BDNF in brain tissue from female mice fed an HFHS diet for 12 weeks [21].

The goal of the current study was to ascertain whether either dietary genistein or moderate-intensity exercise training, or the combination of both, would alter brain markers associated with AD in male mice chronically fed an obesogenic diet consisting of HFHS. To that end, we evaluated physical characteristics, general behavior with open field testing, and expression levels of key markers of AD in brain tissue. Our studies have demonstrated that the incorporation of a diet consisting of 600 mg/kg diet fed to lean healthy mice for 1 month generated serum genistein levels of approximately 4–7 µM [45]. The same serum level of genistein is readily achievable in humans by consumption of soy milk, suggesting that this chosen dose is not only effective but also clinically realistic [46]. Assessing the usefulness of dietary modifications with genistein and/or the inclusion of lifestyle changes such as undertaking regular moderate-intensity exercise training to prevent AD-related pathophysiology that is attributed to diabetes and obesity is crucial given the incidence and prevalence of the population with metabolic syndrome and T2DM. Improved understanding of brain-related disturbances resulting from chronic consumption of Western diets may provide insight for the development of novel targets to alleviate problems associated with Western diet-induced AD. 

## 2. Results

### 2.1. Physical Characteristics

**Figure 1:**
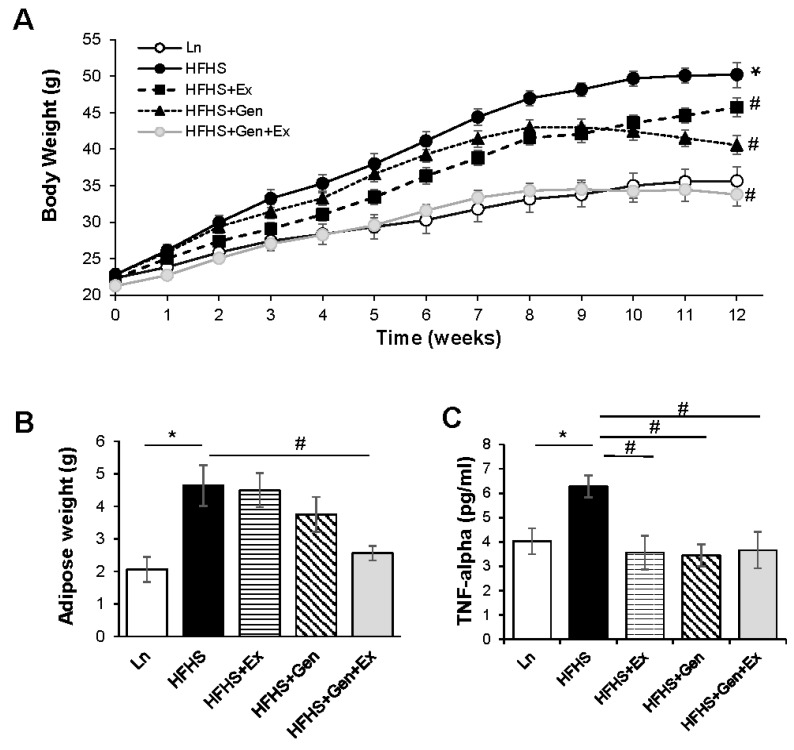
Effect of exercise, genistein, and genistein + exercise combined on HFHS induced changes in physical characteristics. (**A**) Body weight. Weekly measures of body weight taken throughout the 12-week study. Lean, standard diet (open circle, Ln); high-fat high-sugar diet (solid circle, HFHS); HFHS + exercise (solid square, Ex); HFHS + genistein (solid triangle, Gen); HFHS + genistein + exercise (gray circle, Gen+Ex). (**B**) Adipose weight. Abdominal adipose weight measured at completion of the study. (**C**) Serum TNF-α levels. Controls, standard diet (Ln, open bars); high-fat high-sugar diet (HFHS, solid black bars); HFHS + exercise (Ex, horizontal line bars); HFHS + genistein (Gen, hashed bars); HFHS + genistein + exercise (Gen+Ex, gray bars). Data are means ± SEM (n = 7–10/group). * Denotes *p* < 0.05, statistical difference to lean controls, and # denotes *p* < 0.05, statistical treatment effect.

During the 12-week diet study, mice fed the HFHS diet gained significantly more weight (27.34 ± 1.73 g, n = 9, *p* < 0.05) than the lean controls (13.31 ± 1.04 g, n = 10, Figure 1A). All treatments significantly decreased weight compared to HFHS alone, and HFHS+Gen+Ex resulted in the largest decrease in weight gain, such that mice resembled lean controls (12.53 ± 1.44 g, n = 7, *p* < 0.05). The changes in body weight were largely correlated with changes in adipose mass (Figure 1B) and associated with comparable changes in the inflammatory marker TNF-α (Figure 1C).

### 2.2. Evaluation of Key Proteins Involved in Alzheimer’s Disease

**Figure 2:**
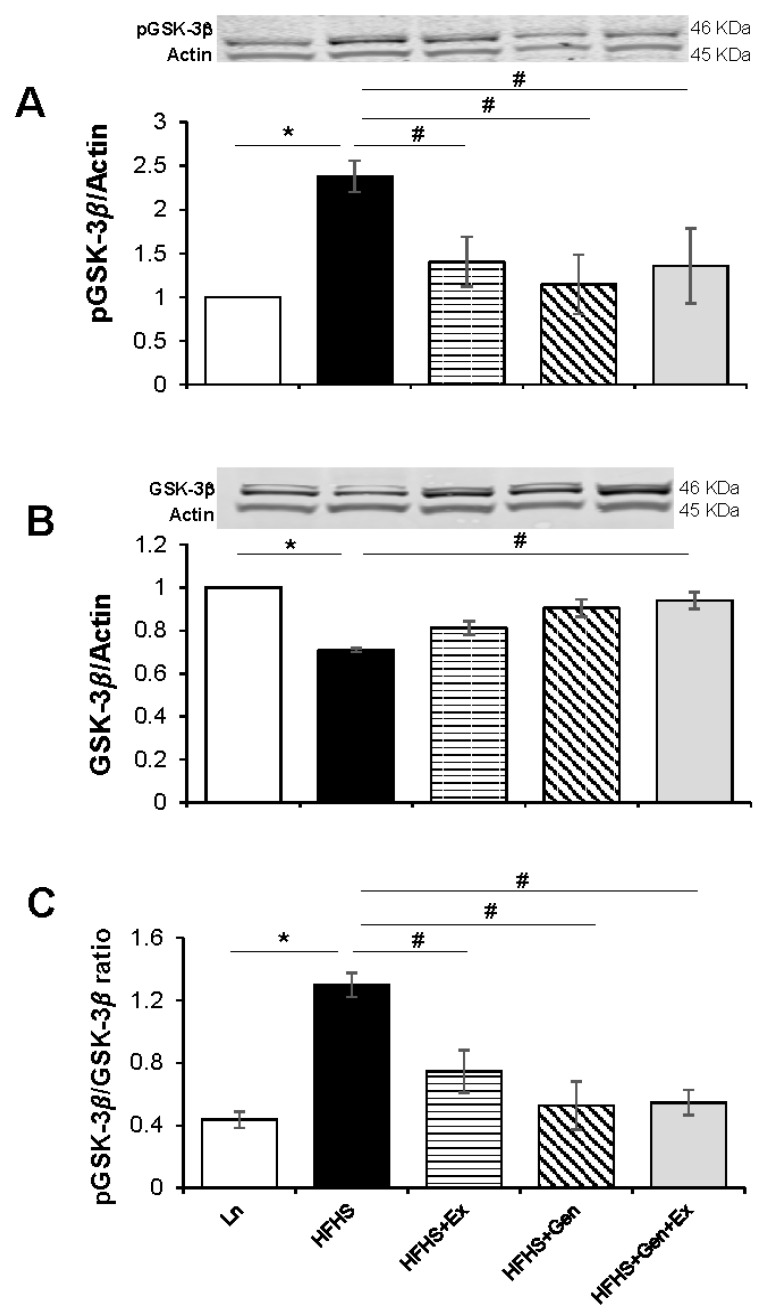
Effect of exercise, genistein, and genistein + exercise combined on expression of pGSK-3β and GSK-3β in male mouse brain. (**A**) pGSK-3β. Representative blot detecting phosphorylated glycogen synthase kinase, pGSK-3B, with average densitometry data. (**B**) GSK-3β. Representative blot detecting glycogen synthase kinase, GSK-3β, with average densitometry data. (**C**) pGSK-3β/GSK-3β ratio. Controls, standard diet (Ln, open bars); high-fat high-sugar diet (HFHS, solid black bars); HFHS + exercise (Ex, horizontal line bars); HFHS + genistein (Gen, hashed bars); HFHS + genistein + exercise (Gen+Ex, gray bars). Data are means ± SEM (n = 4/group). * Denotes *p* < 0.05, statistical difference to lean controls, and # denotes *p* < 0.05, statistical treatment effect.

Total protein expression of several key markers of brain health were evaluated using the standard Western blot technique. Expression of brain phosphorylated glycogen synthase kinase, pGSK-3B, was significantly increased 2.3-fold with HFHS diet (2.37 ± 0.18, n = 4, *p* < 0.05) versus lean controls, and this was significantly mitigated by Ex (1.40 ± 0.29, n = 4, *p* < 0.05), Gen (1.15 ± 0.33, n = 4, *p* < 0.05), and Gen+Ex combined (1.35 ± 0.42, n = 4, *p* < 0.05, Figure 2A). Expression of brain GSK-3B was significantly decreased 0.7-fold with HFHS (0.71 ± 0.01, n = 4, *p* < 0.05) compared to lean controls, and this was significantly mitigated by Gen+Ex combined (0.94 ± 0.04, n = 4, *p* < 0.05, Figure 2B). The ratio of pGSK-3B/GSK shadowed the pGSK-3B expression data and was significantly increased by HFHS diet compared to lean controls, with significant mitigation by Ex, Gen, and Gen+Ex combined (Figure 2C).

**Figure 3:**
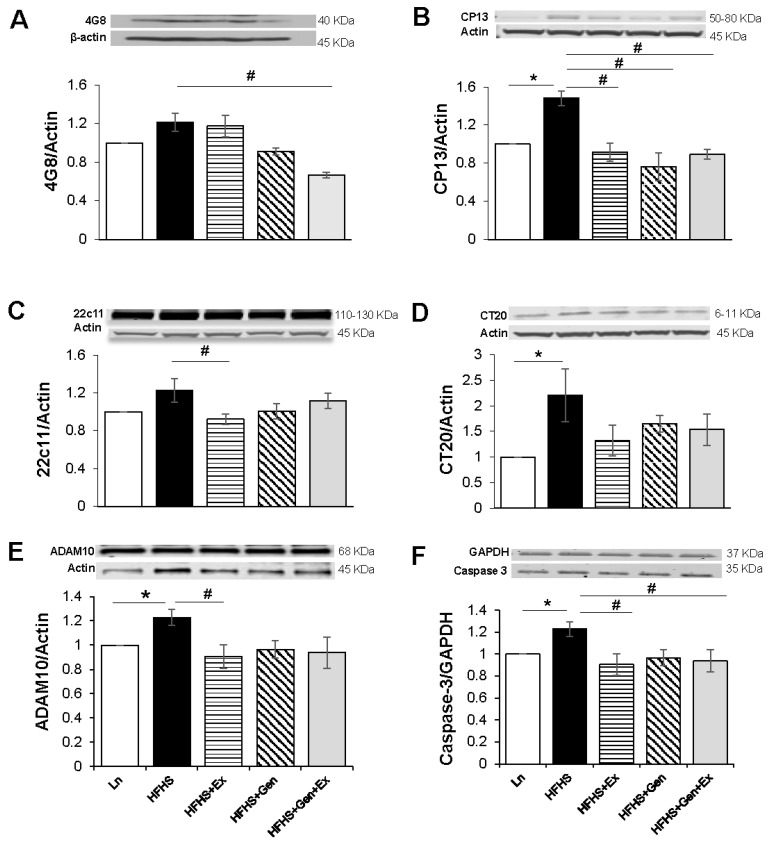
Effect of exercise, genistein, and genistein + exercise combined on expression of 4G8, CP13, 22c11, and CT20 in male mouse brain. (**A**) 4G8. Representative blot detecting Aβ using 4G8, with average densitometry data. (**B**) CP13. Representative blot detecting phosphorylated tau using CP13, with average densitometry data. (**C**) 22c11. Representative blot, with average densitometry data. (**D**) CT20. Representative blot detecting pathological cleavage of Aβ using CT20, with average densitometry data. (**E**) ADAM10. Representative blot detecting A-disintegrin and metalloprotease 10 protein, ADAM10, an alpha-secretase responsible for the non-amyloidogenic pathway of amyloid precursor protein, with average densitometry data. (**F**) Caspase-3. Representative blot detecting apoptosis using caspase-3, with average densitometry data. Controls, standard diet (Ln, open bars); high-fat high-sugar diet (HFHS, solid black bars); HFHS + exercise (Ex, horizontal line bars); HFHS + genistein (Gen, hashed bars); HFHS + genistein + exercise (Gen+Ex, gray bars). Data are means ± SEM (n = 5–6/group). * Denotes *p* < 0.05, statistical difference to lean controls, and # denotes *p* < 0.05, statistical treatment effect.

We evaluated the influence of HFHS, exercise, genistein, and all three together on key brain markers of AD. Quantification of Aβ was assessed using 4G8 expression levels. Expression of 4G8 trended to increase with HFHS diet (1.21 ± 0.09, n = 3) when normalized to lean controls, and was significantly mitigated by Gen+Ex combined (0.67 ± 0.02, n = 3, *p* < 0.05, Figure 3A). CP13 expression (to assess phosphorylated tau) was significantly increased 1.4-fold by HFHS diet (1.48 ± 0.08, n = 4, *p* < 0.05) compared to lean controls and was significantly mitigated by all treatments: Ex (0.92 ± 0.09, n = 5, *p* < 0.05), Gen (0.77 ± 0.15, n = 5, *p* < 0.05), and Gen+Ex (0.89 ± 0.05, n = 5, *p* < 0.05, Figure 3B). Expression of 22c11 (the non-cleaved APP integral membrane protein) trended to increase with HFHS diet compared to lean controls and was significantly mitigated by Ex (0.92 ± 0.05, n = 5, *p* < 0.05, Figure 3C). Expression of CT20 (to detect pathological cleavage of Aβ) was significantly increased 2.2-fold by HFHS diet (2.21 ± 0.51, n = 4, *p* < 0.05) compared to lean controls and was unchanged by treatments (Figure 3D). We quantified expression of A-disintegrin and metalloprotease 10 protein, ADAM10, an alpha-secretase responsible for the non-amyloidogenic pathway of amyloid precursor protein. ADAM10 expression significantly increased 1.2-fold with HFHS diet (1.23 ± 0.07, n = 4, *p* < 0.05) compared to lean controls and was significantly mitigated by Ex (0.91 ± 0.09, n = 5, *p* < 0.05, Figure 3E). To assess whether genistein or exercise (or both) influenced apoptosis, we evaluated caspase-3 expression. Caspase-3 levels were significantly increased 1.2-fold by HFHS diet (1.23 ± 0.07, n = 4, *p* < 0.05) compared to lean controls, and this was mitigated by Ex (0.91 ± 0.09, n = 5, *p* < 0.05) and Gen+Ex combined (0.93 ± 0.10, n = 6, *p* < 0.05, Figure 3F).

**Figure 4:**
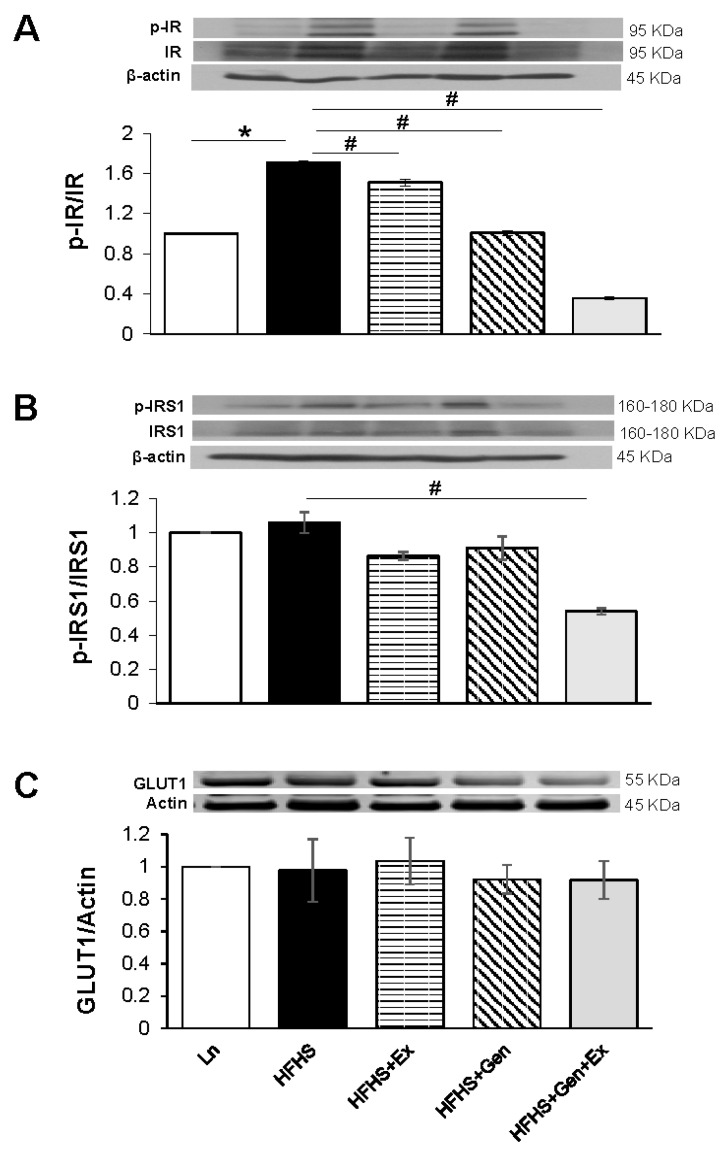
Effect of exercise, genistein, and genistein + exercise combined on expression of pIR/IR, pIRS1/IRS1, and GLUT1 in male mouse brain. (**A**) pIR/IR. Representative blot detecting insulin receptors (phosphorylated/total), with average densitometry data. (**B**) pIR1S/IRS1. Representative blot detecting insulin receptor substrate (phosphorylated/total), with average densitometry data. (**C**) GLUT1. Representative blot detecting glucose transporter 1, GLUT1, with average densitometry data. Controls, standard diet (Ln, open bars); high-fat high-sugar diet (HFHS, solid black bars); HFHS + exercise (Ex, horizontal line bars); HFHS + genistein (Gen, hashed bars); HFHS + genistein + exercise (Gen+Ex, gray bars). Data are means ± SEM (n = 4/group). * Denotes *p* < 0.05, statistical difference to lean controls, and # denotes *p* < 0.05, statistical treatment effect.

To better understand the insulin sensitivity of the brain tissue from HFHS-fed mice and to assess the effects of genistein and exercise thereon, we evaluated the expression of insulin receptors (phosphorylated/total), and insulin receptor substrate (phosphorylated/total). The ratio of pIR/IR was significantly increased 1.7-fold with HFHS diet (1.04 ± 0.01, n = 3, *p* < 0.05) compared to lean controls (0.61 ± 0.00, n = 3, Figure 4A). All treatments significantly decreased the pIR/IR ratio (Figure 4A). We found no change in the phosphorylated insulin receptor substrate-1 (IRS1) compared to total IRS1 expression with HFHS diet; however, Gen+Ex combined reduced the ratio (Figure 4B). GLUT1 expression was unchanged by HFHS diet and, moreover, remained unmodified by all treatments (Figure 4C).

### 2.3. Behavioral Assessments

**Figure 5:**
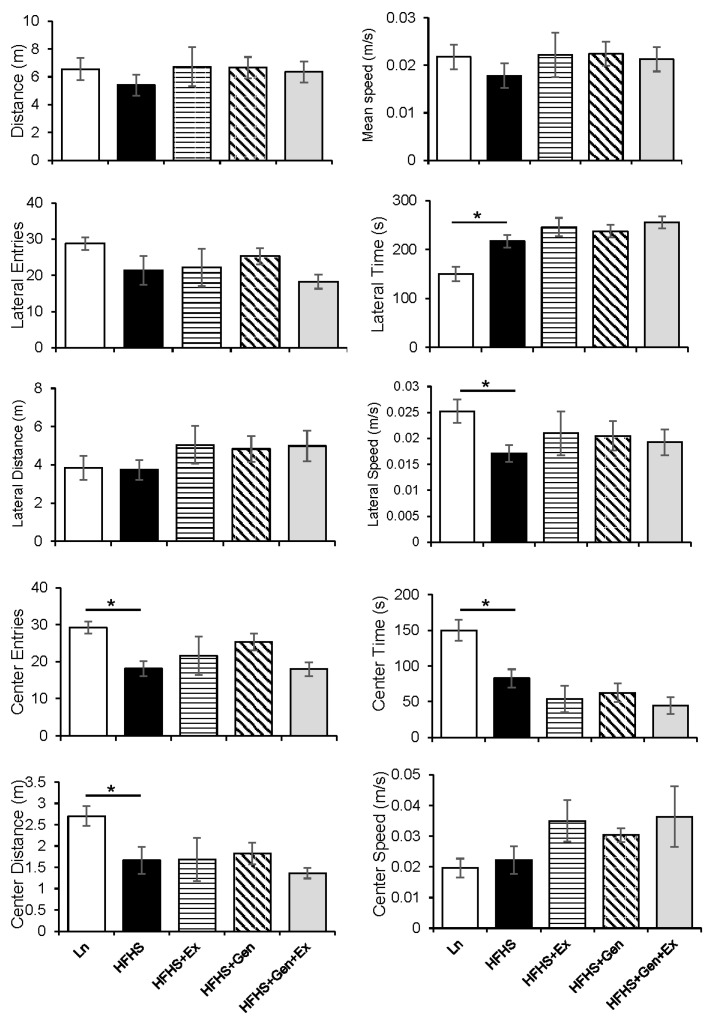
Effect of exercise, genistein, and genistein + exercise combined on open-field behavioral testing in male mice. Quantification of the time the mice spent in lateral spaces and center areas with distance covered in each and average speed in each space. Controls, standard diet (Ln, open bars); high-fat high-sugar diet (HFHS, solid black bars); HFHS + exercise (Ex, horizontal line bars); HFHS + genistein (Gen, hashed bars); HFHS + genistein + exercise (Gen+Ex, gray bars). Data are means ± SEM (n = 4–5/group). * Denotes *p* < 0.05, statistical difference to lean controls.

Quantification of open field recordings suggested that mice fed an HFHS diet spent more time in the outer area of the box (lateral time), and while in the outer lateral area had a slower speed compared to lean controls. Moreover, treatments were without effect (Figure 5). Mice fed an HFHS diet entered the center space significantly less frequently (21.40 ± 3.97 versus 29.25 ± 1.62, *p* < 0.05), spent less time in the center space (82.76 ± 12.65 s versus 150.09 ± 14.66 s, *p* < 0.05), and moved less distance in the center (1.66 ± 0.31 m versus 2.70 ± 0.24 m, *p* < 0.05) compared to lean counterparts. Treatments of either exercise or genistein or both were without effect on behavioral testing.

## 3. Discussion 

The use of hypercaloric diets rich in saturated fats, simple sugars, or a combination thereof to induce obesity in rodents is a well-established model to understand the underlying etiologies and consequences of obesity-related disorders. Energy-rich diets promote obesity by increasing adipose tissue weight, leptin and insulin resistance, disturbances in glucose metabolism, and the incidence of type 2 diabetes. Consumption of hypercaloric foods also increases the risk of developing age-related brain disorders, including cognitive dysfunction, dementia, and AD-related neuropathology. Indeed, adherence to a poor diet and physical inactivity converge to contribute to the pathogenesis of late-onset AD [47]. Despite recent advances in drug development for the treatment of cognitive decline and AD, there remains no effective treatment. Lifestyle interventions or modifications combining physical activity with wholesome eating and decreasing the consumption of refined foods remain effective forms of nonpharmacological therapy to promote overall health outcomes. Several studies indicate that regular exercise delays the progression of obesity-related metabolic disorders and neurodegenerative diseases, including AD. Furthermore, natural bioactive compounds such as the isoflavone genistein, which is abundant in plant foods, are also beneficial due to their anti-inflammatory, antioxidant, and neuroprotective effects [48,49]. In the present study, we investigated the effects of genistein supplementation, moderate-intensity exercise, and the combination of both on HFHS-induced brain neuropathology. 

Our results demonstrate that after 12 weeks of HFHS feeding, as expected, mice developed obesity with significant adipose tissue weight gain and systemic inflammation. The metabolic consequences of this diet on the same mouse model were recently reported and, notably, were accompanied by visceral obesity, hyperleptinemia, hyperglycemia, and systemic insulin resistance [17]. A pathophysiological association between insulin resistance and AD has been suggested; emerging evidence indicates that defective insulin signaling in the brain is associated with the development of cognitive dysfunction and neuropathology, including the accumulation of toxic species of Aβ, pretangles, and mature NFTs, all of which are hallmarks of AD [50]. Aberrant brain insulin signaling is the phosphorylation of tau protein and the production of senile plaques and Aβ peptide deposits, a process mediated through activation of GSK-3β [51]. By virtue of its decisive kinase-dependent functions, levels of phosphorylated GSK-3β (pGSK-3β) were measured in this study as a key marker to detect HFHS-induced aberrations in brain metabolism. This enzyme favors the production of senile plaques and deposition of Aβ peptides [52]. Consistent with the changes induced by elevated pGSK-3β levels, insulin resistance in brain tissue of these HFHS-fed obese mice was confirmed by the presence of elevated p-IR/IR levels. Further, we found increased levels of phosphorylated tau, detected by CP13, which indicated the development of both pretangles and mature NFTs. In addition, we detected elevated levels of amyloid precursor protein (APP using CT20) in the brain following HFHS feeding. Taken together, our analysis of brain tissue from mice fed an HFHS diet revealed significant aberrations induced by pGSK-3β that are consistent with central insulin resistance and AD pathology [53,54]. These findings mirror earlier studies using the HFHS and diabetic models and are also reminiscent of the human condition [55,56]. In addition to the consequences on insulin resistance, a connection between pGSK-3β, neuroinflammation, and apoptosis has been recognized [57]. In line with this association, we show that the increased expression of pGSK-3β was accompanied by apoptosis, expressed as ADAM10 [58] and caspase-3. Given that plasma inflammatory cytokines, including TNF-α, are able to cross the blood–brain barrier and induce neuroinflammation and accelerate neurodegeneration [59,60], it is conceivable that they do so in this model.

Population-based studies have demonstrated correlations between high intake of saturated fats and/or sugars to increased risk of cognitive dysfunction, dementia, AD, and mortality rates [32,61,62,63]. Preventative interventions aimed at decreasing the risk of cognitive dysfunction include simple lifestyle modifications such as regular exercise, healthy eating, or effectively combining both [33], and are known to improve overall neurological health. The neuroprotective effects of the plant-based soy-rich isoflavone genistein have been extensively studied in rodent models of obesity, insulin resistance, and severe diabetes [20] and in human studies [64,65,66]. The beneficial effects of genistein consumption on brain health and cognitive function have been attributed to improved endothelial function and cerebral blood flow [67], and metabolically, to genistein-mediated antioxidant, anti-apoptotic, and anti-inflammatory actions [48]. In the present study, genistein treatment reduced weight gain and systemic inflammation in HFHS-fed mice, and reduced expression of pGSK-3β and pIR/IR, indicating improvements in insulin sensitivity in the brain. This is consistent with recent studies showing enhanced cerebral glucose homeostasis through upregulation of glucose transporter mechanisms in the aging rat brain [68]. Chronic genistein treatment is also known to improve insulin action in peripheral tissues, resulting in a glucose-lowering effect in obesity [17]. Consistent with this effect on glucose homeostasis, systemic reviews examining the role of genistein as a potential intervention for diabetes suggest an inverse relationship between genistein consumption and the risk of developing prediabetes [69,70]. Our data further demonstrated that reduced expression of pGSK-3β in genistein-fed mice was associated with a decreased phosphorylated tau (CP13), which detects it at an early nonfibrillary state and later in neurons containing NFTs. Our findings are consistent with a recent study showing a neuroprotective effect of chronic genistein treatment against neural degeneration in the ApoE^−/−^ mice fed an obesogenic diet [26]. While most studies report neuroprotective effects following long-term treatment, benefits were also reported following short-term treatment (4-weeks) with genistein in the *ob/ob* mouse, whereby genistein prevented toxic amyloid species load and enhanced the expression of NGF and BDNF [20], highlighting the neuroprotective efficacy of genistein. 

While current drug treatments are ineffective in reversing AD-like neuropathology, regular exercise is a well-recognized and effective non-pharmacological approach known to delay cognitive function and the progression of neurodegenerative diseases by reducing inflammation, Aβ content, and apoptosis, and stimulating neurogenesis [37,40,41,42,43,44]. Herein, the benefits of regular moderate-intensity exercise training and neuroprotection are further supported by our results. We show that exercise training provided neuroprotection in HFHS-fed mice at a level that was slightly superior to that of genistein-feeding. Systemic inflammation, markers of brain insulin resistance, and phosphorylated-tau load were equally reduced with exercise and genistein treatment. However, decreased apoptosis, detected by ADAM10 and caspase-3, and reduced APP, detected by 22c11 (involved in the production of Aβ), were added benefits of the exercise treatment compared to genistein alone. The mechanisms behind exercise-induced neuroprotection on these markers of AD have yet to be determined, but recent studies have reported that, in addition to increasing the expression of BDNF [21], exercise reduces both the levels of autophagy markers [40] and the expression of beta-secretase 1 (BACE1) [71,72]. Aβ is formed by APP after processing by BACE1, and an increase in BACE1 expression, which was previously reported in mice fed an HFHS diet [55], indicates processing of APP and generation into Aβ. Levels of BACE1 were not measured in this study, but the reduction in APP detected by 22c11 following exercise training suggests decreased production or increased clearance of Aβ though other pathways. Decreased toxic species of Aβ and reduced expression of BACE1 levels were reported in the 3x-Tg model of AD after treadmill running [40,42,43,73,74]. Clearly, regular exercise impacts brain health, and evidence suggests that the benefits on the brain are not exercise paradigm specific. In this study, we chose to exercise train at moderate intensity, which allowed the mice to run without reluctance or difficulty from their obesity, thus completing the training protocol. Decreased Aβ load, phosphorylated tau levels, and neuroinflammation are known to occur with other forms of exercise, such as voluntary wheel running [75], resistance training [74], and high-intensity interval training, in T2DM rats [76]. This suggests that exercise training, regardless of the form, affords neuroprotection against the effects of HFHS feeding and in models of AD. Interestingly, we found that HFHS feeding induced some behavioral changes (decreased center entries, deceased center time, and decreased center distance), perhaps indicative of a reduction in exploratory behavior. However, all interventions were without effect on these HFHS-induced behavioral changes, suggesting that interventions may mitigate brain biochemical pathways without modifying behavioral changes. 

## 4. Materials and Methods

### 4.1. Mouse Model of Diet Induced Diabetic Obesity

Male C57BL/6 mice (4 weeks old) were purchased from Charles River Laboratory (Wilmington, MA, USA). Following a 1-week acclimation period, the 45 mice were randomly divided into five groups for a period of 12 weeks: standard rodent diet Harlan 5001 (Std, n = 10) with normal water, high-fat diet with high-sugar water (HFHS, 60% fat, n = 9), high-fat diet with high-sugar water + genistein (HFHS+Gen, 600 mg genistein/kg high fat diet, n = 9), high-fat diet with high-sugar water + exercise (HFHS+Ex, n = 10), high-fat diet with high-sugar water + genistein + exercise (HFHS+Gen+Ex, n = 7). All mice fed the high-fat diet were also exposed to sugar in the drinking water (42 g/L water; 55% fructose/45% sucrose) throughout the 12-week diet study. Food and water were provided ad libitum. The high-fat diet, with or without genistein, was purchased from Dyets Inc. (Bethlehem, PA, USA). The exercise regimen comprised running mice on a treadmill 5 days/week, for a total of 150 min/week from week 3 to 12, as previously described [77]. We utilized a moderate-intensity exercise protocol as follows: week 1 (10 min at 10 m/min), week 2 (20 min at 10 m/min), week 3 (30 min at 12 m/min), weeks 4–12 (30 min at 15 m/min). Mice were housed two per cage with a 12:12 h light–dark cycle. Body weight and overall health were assessed each week. At the end of the 12-week study, the mice were euthanized by asphyxiation in 100% CO_2_, followed immediately by surgical thoracotomy (inducing pneumothorax). Blood was immediately collected via cardiac puncture and centrifuged and stored at −80° until use. Animal care was conducted in accordance with established guidelines, and all protocols were approved by the Midwestern University Institutional Animal Care and Use Committee (MWU-IACUC #3019, approved 18 November 2020). 

### 4.2. Behavioral Assessments

The mice were acclimated to an open field box (16″ by 16″) and two × five-minute recordings/mouse were analyzed using Any-Maze Software 7.4 (Stoelting Co., Wood Dale, IL, USA). Per prior studies, distance, speed, and entries were assessed in both the center and the lateral space of the box [78].

### 4.3. Western Blot Analysis for Total Protein Expression

At euthanasia, brains were immediately snap frozen in liquid nitrogen and stored at −80 °C. Brain tissue was later prepared for Western blot analysis, as described previously [21]. Blots were loaded with 5 ug protein/lane and individually incubated with each of the following primary antibodies overnight at 4 °C: pGSk-3ß (1:1000, Cell Signaling #5558, Danvers, MA, USA), GSK-3ß (1:1000, Cell Signaling #9315, Danvers, MA, USA), Caspase-3 (1:1000, Cell Signaling #9662, Danvers, MA, USA), 4G8 (1:500, BioLegend #SIG-39200, San Diego, CA, USA), CP13 (1:1000, Cell Signaling #39357, Danvers, MA, USA), 22c11 (1:1000, Millipore #MAB348, St. Louis, MO, USA), CT20 (1:1000, Millipore #171610, St. Louis, MO, USA), ADAM10 (1:1000, Cell Signaling #14194, Danvers, MA, USA), p-IR (1:1000, Cell Signaling #3026, Danvers, MA, USA), IR (1:500, Santa Cruz #sc-373975, Dallas, TX, USA), pIRS1 (1:1000, Cell Signaling #2386, Danvers, MA, USA), IRS1 (1:1000, Millipore-Sigma #06-248, Burlington, MA, USA), GLUT1 (1:500, Alomone #AGT-021, Jerusalem, Israel). Blots were either re-probed for actin (anti-actin primary antibody, 1:4000, Thermo Scientific, Rockford, IL, USA) or re-probed for GAPDH (anti-GAPDH primary antibody, 1:4000, Thermo Scientific, Rockford, IL, USA) for 1 h at room temperature to serve as the control. Blots were washed and then incubated with the appropriate secondary antibodies—anti-mouse IgG (H+L) (1:15,000, Dylight, Thermo Scientific, Rockford, IL, USA) and anti-rabbit IgG (H+L) (1:15,000, Dylight, Thermo Scientific, Rockford, IL, USA)—for 1 h at room temperature. Images of membranes were taken with all proteins of interest normalized to either actin or GAPDH. Band density was analyzed using Odyssey-Clx (LI-COR, Lincoln, NE, USA) and Image Studio (LI-COR, Lincoln, NE, USA). For all blots, lean controls were taken as 1, and all other groups were compared to lean controls.

### 4.4. Serum Measures

Serum samples were assayed for TNFα (Milliplex Mouse Cytokine Bead Panel, EMD Millipore Corporation, Billerica, MA, USA). 

### 4.5. Statistics

Data are expressed as mean ± SEM. Numbers in parentheses represent the number of tissues used from separate individual mice. One-way ANOVA with Dunnett’s multiple comparisons test was performed using GraphPad (https://www.graphpad.com/, San Diego, CA, USA) to compare lean controls to HFHS mice and assess the effect of treatment versus HFHS alone, and *p* < 0.05 was considered statistically significant.

## 5. Conclusions

In conclusion, we show that the HFHS mouse model of obesity exhibited aberrations in protein levels that are consistent with neurodegeneration and linked to AD pathology. The most effective approach providing the greatest protection was combining exercise training with genistein treatment. Combination treatment in the presence of chronic HFHS feeding prevented adipose tissue mass gain and inhibited the development of obesity, exerted a robust effect on brain insulin sensitivity, and decreased the deposition of protein levels of Aβ, detected by the antibody 4G8. The added benefits of combined treatment in this model of diet-induced obesity are consistent with our recent studies examining the impact of this form of treatment on neurodegeneration in the female mouse brain [21]. Combined exercise training and genistein mitigated the unwanted effects of obesity on insulin action and glucose homeostasis, hepatic gluconeogenesis, hepatic injury, and steatosis [17]. The results of our study highlight the importance of regular moderate-intensity exercise and consumption of isoflavone genistein to delay neurodegeneration of the brain.

## Data Availability

The data generated during this study are available upon request.

## Abbreviations

TNF-α	Tumor necrosis factor-alpha
Aβ	Amyloid beta
pGSK-3β	Phosphorylated glycogen synthase kinase
GSK-3β	Glycogen synthase kinase
APP	Amyloid precursor protein
NFT	Neurofibrillary tangles
BDNF	Brain-derived neurotrophic factor
NGF	Nerve growth factor
HFHS	High-fat high-sugar
